# Adsorption of As(V) from Aqueous Solution on Chitosan-Modified Diatomite

**DOI:** 10.3390/ijerph17020429

**Published:** 2020-01-08

**Authors:** Qintao Yang, Liang Gong, Lili Huang, Qinglin Xie, Yijian Zhong, Nanchun Chen

**Affiliations:** 1 College of Environmental Science and Engineering, Guilin University of Technology, Guilin 541006, China; BX2018024@glut.edu.cn (Q.Y.); 2120180304@glut.edu.cn (L.G.); ye.llowlili@163.com (L.H.); zhongyijian@glut.edu.cn (Y.Z.); 2 Guangxi Key Laboratory of Theory and Technology for Environmental Pollution Control, Guilin University of Technology, Guilin 541006, China; 3 Collaborative Innovation Center for Water Pollution Control and Water Safety in Karst Area, Guilin University of Technology, Guilin 541006, China; 4 College of Materials Science and Engineering, Guilin University of Technology, Guilin 541004, China; cnc@glut.edu.cn

**Keywords:** CS-modified Dt, As(V) ions, removal efficiency

## Abstract

A novel chitosan (CS)-modified diatomite (Dt) was prepared by a simple mixture in the mass ratio to remove As(V) from aqueous solution in this research. The CS-modified Dt adsorbent was characterized by scanning electron microscopy (SEM), Fourier-transform infrared spectroscopy (FTIR), and X-ray powder diffraction (XRD) analysis. The parameters to influence the adsorption of As(V) ion were studied under such conditions as kinetics, adsorption isotherm, and pH effect. The results revealed that adsorption of As(V) was initially rapid and the equilibrium time was reached after 40 min. The optimal value of the pH was 5.0 for better adsorption. The equilibrium data were well fitted to the Langmuir isotherm compared to the Freundlich isotherm, and exhibited the highest capacity and removal efficiency of 94.3% under an initial As(V) concentration of 5 mg/L. The kinetic data were well described by the pseudo-second-order model. In addition, 0.1 M NaOH has the best desorption efficiency of As(V) adsorbed on CS-modified Dt, and the removal efficiency of As(V) was still higher than 90% when after six adsorption-desorption cycles. These results showed that the CS-modified Dt could be considered as a potential adsorbent for the removal of As(V) in aqueous solution.

## 1. Introduction

Water pollution is one of the most critical environmental problems at present, especially those involving heavy metal contamination which can adversely affect humans and animals [1,2,3]. In an aquatic environment most arsenic species are present as an inorganic oxyanion, namely arsenite (AsO_3_^3−^ or As(III)) and arsenate (AsO_4_^3−^ or As(V)), which are more toxic and harmful than its organic forms [4]. Under the oxidizing condition, As(III) is unstable and can be oxidized to As(V) [5]. Long-term arsenic exposure can result in serious threats to human health including cardiovascular disease, hepatosis, and even cancer [6]. Therefore, the World Health Organization have suggested the permissible limit of arsenic in drinking water as 0.01 mg/L while the permissible limit of arsenic in wastewater from industries restricted by the Ministry of Ecology and Environment of the People’s Republic of China is 0.5 mg/L before releasing to the environment. Owing to its hazard, the removal of inorganic As(V) from aqueous solution is very urgent.

Up to now, many treatment methods such as coagulation-flocculation [7], oxidation-precipitation [8], membrane filtration [9], ion exchange [10], and adsorption methods [11] have been applied to remove inorganic arsenic from water. Over the past decades, adsorption has become one of the most efficient and economical techniques among those methods [12]. Therefore, the research of low-cost adsorbents for arsenic removal from wastewater has attracted more interest.

Chitosan (CS) is a natural polymer compound, which possesses non-toxicity, biocompatibility, and biodegradation properties [13]. Due to the presence of active functional groups such as hydroxyl, amino groups, and glycosidic bond, CS displays a powerful adsorption performance toward heavy metal ions from aqueous solutions [14,15,16,17,18]. However, pure CS as an adsorbent has several disadvantages, including high cost and low chemical stability, which limits the further application of CS in wastewater treatment [19]. Immobilizing CS on a low-cost clay such as montmorillonite [20], bentonite [21], sand [22], perlite [23], and ceramic alumina [24] could reduce the required amount of CS without affecting the overall adsorption capacity. The CS-modified clay, therefore, has a practical use as a potential material to serve as a permeable reactive barrier in the treatment of contaminated surface water. Diatomite (Dt) is a siliceous rock made up largely from diatoms, which has a large number of natural, ordered pores and large specific surface area [25,26]. It is abundant in many areas such as China, the United States, Japan, Denmark, France, and Romania, and has a lower cost than CS. Therefore, Dt is an attractive immobilization material for CS owing to its low cost as well as its chemical and mechanical stability.

Hence the study focused on the modification Dt by Cs for As(V) removal from the aqueous solution. The structural and chemical characterization of CS-modified Dt were studied by various techniques such as SEM, FTIR, and XRD analysis. As(V) sorption experiment was studied by optimizing various adsorption parameters like contact time, solution pH, temperature, and dosage of adsorbents in batch mode. The adsorption isotherms and kinetics of As(V) ion adsorption for the CS-modified Dt were studied, and the desorption and reusability of the CS-modified Dt for adsorption As(V) were also investigated.

## 2. Materials and Methods 

### 2.1. Materials

The raw Dt (92.8% SiO_2_, 4.2% Al_2_O_3_, 1.5% Fe_2_O_3_, and other metal oxides) were purchased from Jilin Kaida Diatomite Co. Ltd (China). CS (95% deacetylated) was purchased from Shanghai Aladdin Bio-chem Technology Co. Ltd (China). Sodium arsenate (Na_3_AsO_4_·12H_2_O) was obtained from Shanghai Chemical Reagent and Supply Station (China). All other chemical reagents were analytical grade and were thus used without further purification.

### 2.2. Preparation of CS-Modified Dt

The CS-modified Dt was prepared according to the report by Zhao [27]. Chitosan (CS) powder (2.5 g) was dissolved in 50 mL of 4% (*v*/*v*) acetic acid solution, and then Dt (5 g) was added and stirred until the mixture was homogeneous and gel-like. The CS-modified Dt was dried in an oven at 378 K for 4 h. After drying, the final product was ground and sieved using a 100 mesh sieve and then sealed as CS-modified Dt.

### 2.3. Characterization

The surface morphology was observed by SEM (Hitachi S-4800, Hitachi Company, Japan). The functional groups of Dt and CS-modified Dt were characterized by FTIR (Nexus 470, Thermo Nicolet, USA). The XRD (X’Pert PRO, PANalytical B. V, Netherlands) analysis was performed with Cu K_α_ radiation (λ = 0.154 nm) on a Rigaku X-ray diffractometer. BET (Brunauer–Emmett–Teller) surface area and BJH (Barrett–Joyner–Halenda) pore size distribution were measured by a specific surface analyzer (Micromeritics ASAP 2020, Norcross, GA, USA). The zeta potential of adsorbent was measured by Zetasizer Nano-Zeta potential analyzer (Malvern Instruments, Malvern, UK). X-ray photoelectron spectroscopy (XPS) measurements were taken with a VG Escalab 250Xi (Thermofischer, MA, USA) spectrometer equipped with an Al-Kα = 1486.7 eV). The concentration of arsenic in samples was measured by AFS-9130 atomic fluorescence spectrometry (AFS) (Beijing Jitian, China).

### 2.4. Batch Adsorption Experiments

Batch adsorption experiments were conducted using the CS-modified Dt as an adsorbent. To determine the optimum values for sorbent dosage, pH of solution, contact time, and temperature, a series of preliminary experiments was performed. The specific experimental operation is as follows: 0.01–0.06 g CS-modified Dt was added to 100 mL of 5 mg/L solution of As(V). The pH of the solution was adjusted from 2 to 9 by 0.01 M HCl or NaOH solution, with contact time varying from 1 to 120 min and shaking at different temperatures (298 K, 308 K, and 318 K). The adsorption amounts and the removal efficiency were calculated by the following equation:

(1) $$Q_{t}=\frac{\left(C_{0}-C_{t}\right)V}{m},$$

(2) $$Q_{e}=\frac{\left(C_{0}-C_{e}\right)V}{m},$$

(3) $$\mathrm{Remove}\;\mathrm{efficiency}\;\left(\%\right)=\frac{C_{0}-C_{e}}{C_{0}}\times 100,$$

where, *Q_t_*, *Q_e_* (mg/g) are adsorption capacity at time t and equilibrium, *C_0_*, *C_t_*, *C_e_* (mg/L) are initial, time t and equilibrium concentrations of As(V), respectively, *V* (L) is volume of the aqueous solution, and *m* (mg) is the mass of adsorbent used in experiments.

To describe the results of As(V) ions binding onto CS-modified Dt, Langmuir and Freundlich isotherm models were adopted. The two isotherm models are typically expressed as follows:

(4) $$C_{e}/Q_{e}=C_{e}/Q_{m}+1/K_{L}Q_{m},$$

(5) $$\ln Q_{e}=\ln K_{F}+1/n\ln C_{e},$$

where *Q_e_* (mg/g) and *C_e_* (mg/L) express the adsorption capacity of CS-modified Dt and the equilibrium concentration of the As(V) in the solution, *Q_m_* (mg/g) is the maximum amount of the As(V) adsorbed, *K_L_* is Langmuir adsorption coefficient, *K_F_* is Freundlich adsorption coefficient, and n is Freundlich empirical constant.

To further examine the controlling mechanism of adsorption of As(V) ions onto CS-modified Dt, pseudo-first-order (PFO) and pseudo-second-order (PSO) kinetic models were used to test the obtained experimental data. The equations are given below:

(6) $$\ln\left(Q_{e}-Q_{t}\right)=\ln Q_{e}-K_{1}t,$$

(7) $$t/Q_{t}=1/\left(K_{2}Q_{e}^{2}\right)+t/Q_{e},$$

where *Q_t_* (mg/g) and *Q_e_* (mg/g) are the amount of As(V) adsorption on the CS-modified Dt at *t* (min) and equilibrium, respectively, *K*_1_ and *K*_2_ are the PFO and the PSO rate constant, respectively.

### 2.5. Desorption and Regeneration Studies

Adsorbent regeneration was conducted as follows: 100 mL of 5 mg/L solution of As(V) was contacted with 0.04 g of CS-modified Dt for 40 min at 298 K and pH 5. The mixture was filtered and adsorbed As(V) analyzed. Loaded adsorbents were thoroughly washed with deionized water, filtered and transferred to bottles containing desorbing agent (0.1 M NaOH, 0.1 M NaHCO_3_, 0.1 M NaCl, 0.1 M HCl, and 0.1 M CH_3_COOH) and shaken for 12 h at 298 K. A solution of 0.1 M NaOH was applied for adsorbent regeneration and the regeneration of CS-modified Dt was sequentially operated six adsorption-desorption cycles. Desorption ratio was calculated from the following equation:

(8) $$\mathrm{Desorption}\;\mathrm{efficiency}\;\left(\%\right)=\frac{\left(\mathrm{As}\left(V\right)\mathrm{desorbed}\right)}{\left(\mathrm{As}\left(V\right)\mathrm{adsorbed}\right)}\times 100.$$

## 3. Results and Discussion

### 3.1. FTIR Analysis

**Figure 1:**
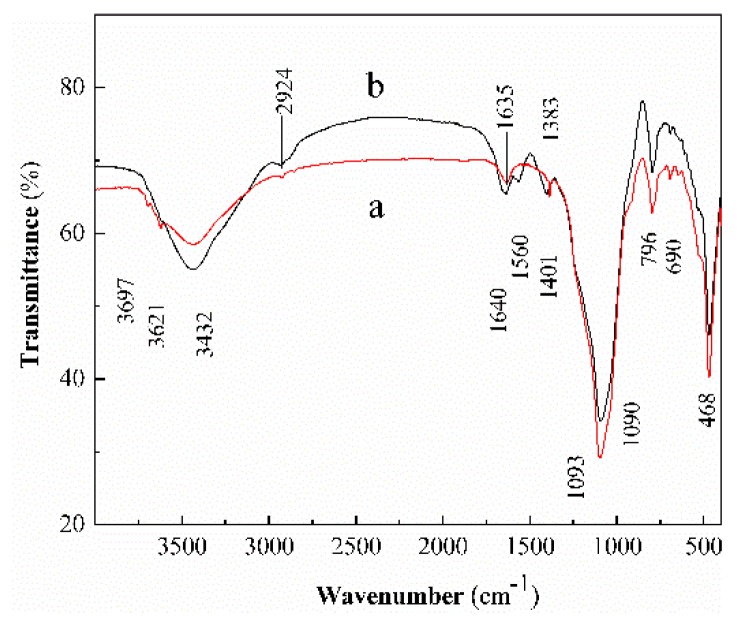
The FTIR spectra of (**a**) raw diatomite and (**b**) chitosan (CS)-modified diatomite (Dt).

Figure 1 shows the FTIR spectra of the raw Dt and CS-modified Dt. In Figure 1a, the main spectral peaks of functional groups are detected at 3697, 3621, 3432, 1635, 1383, 1090, 796, 690, and 468 cm^−1^. The peaks at 3697, 3621, and 3432 cm^−1^ are corresponding to the stretching vibrations of Al-OH, Si-OH, and H-OH [28,29]. The peak at 1635 cm^-1^ is due to the bending vibration of H–OH [30]. The peak at 1383 cm^−1^ is likely caused by the bending vibration of C–H [31]. The peak at 1090 cm^−1^ is attributed to the anti-symmetrical stretching vibration of Si–O–Si [32]. The peaks at 796, 690, and 468 cm^−1^ are the result of bending vibration by Si–O [30,33]. In Figure 1b, the peaks at 3697 and 3621 cm^−1^ are disappeared and the peak at 3432 cm^−1^ is broadened and enhanced, resulting from overlapping between the N–H and –OH stretching vibrations [27]. Compared to the spectrum of the raw Dt, there is a significant difference, with two new peaks appearing in the spectrum of the CS-modified Dt at 2924 and 1560 cm^−1^ attributed to –CH_2_- stretching vibration and –NH_2_ bending vibration of the CS [34,35]. The peaks at 1635, 1090, and 1383 cm^−1^ were shifted to 1640, 1093 and 1401 cm^−1^, respectively. These changes could be attributed to a hydrogen bond interaction between Dt and CS [36]. Therefore, we speculate that Dt is successfully modified with CS [37].

**Figure 2:**
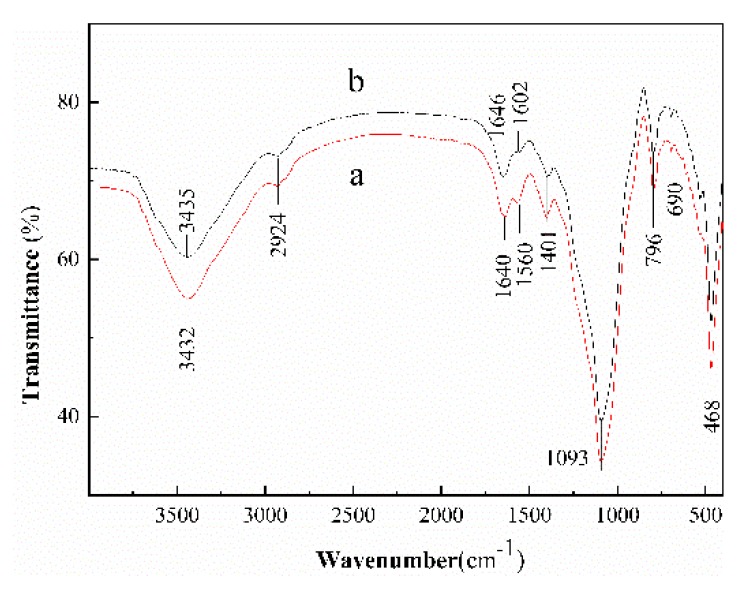
The FTIR spectra of (**a**) CS-modified and (**b**) CS-modified Dt after adsorption.

Figure 2 shows the FTIR spectra of the CS-modified Dt and CS-modified Dt after adsorption. Comparing Figure 2a,b, the peak at 3432 cm^−1^ was shifted to 3435 cm^−1^, the peak at 1640 cm^−1^ was shifted to 1646 cm^−1^, the peak at 1560 cm^−1^ was shifted to 1602 cm^−1^. It indicates that the adsorption of As(V) ions on the surface of CS-modified Dt are either through complexation or through the physical way which might be through weak electrostatic interaction and Van der Waals forces [38].

### 3.2. SEM Images

**Figure 3:**
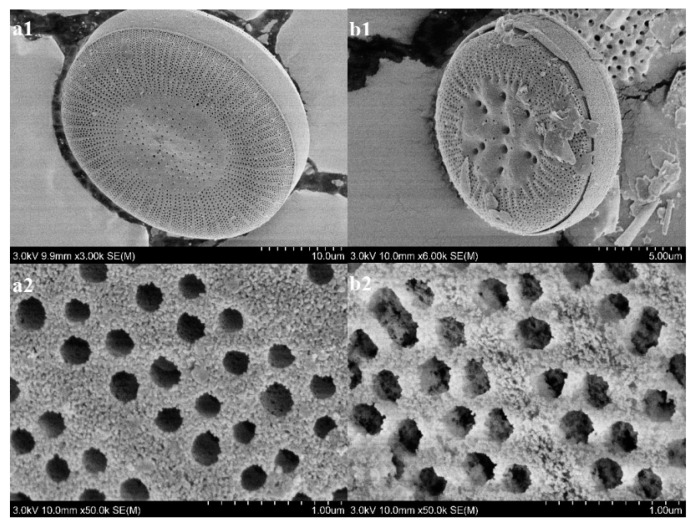
SEM images of raw Dt (**a1**,**a2**) and CS-modified Dt (**b1**,**b2**).

The SEM images of the raw Dt and CS-modified Dt samples are presented in Figure 3. As can be seen in Figure 3(a1), Dt morphology was clearly visible; the shape was mainly a disk structure similar to that of a ceramic membrane. Its surface was smooth and flat, with abundant porous structures (Figure 3(a2)). As illustrated in Figure 3(b1), the CS attached to the surface of the Dt did not change the porous structure of Dt. Moreover, surface of CS-modified Dt was rough presented in Figure 3(b2). These findings indicated that Dt was successfully combined with Dt.

### 3.3. BET Analysis

**Figure 4:**
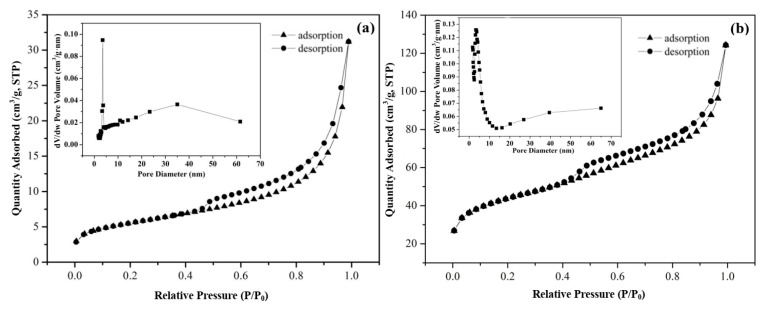
N_2_ adsorption/desorption isotherms of (**a**) raw Dt and (**b**) CS-modified Dt (Inserts are pore diameter distributions).

According to the classification curve proposed by the IUPAC for adsorption isotherms, the isothermal curves of raw Dt and CS-modified Dt, illustrated in Figure 4 of (a) and (b) belonged to a type IV cure. The adsorption capacities of the two curves presented in the diagram were not equal to 0 in the extremely low-pressure range (P/P_0_ < 0.01), and the increase of pressure promoted the adsorption capacity of CS-modified Dt to a greater degree than that of Dt. This outcome showed that both of them had micropore structures [39], and those of CS-modified Dt were more abundant because of modification that had occurred. As can be seen in Table 1, the average pore diameter of raw Dt and CS-modified Dt were 9.998 nm and 5.185 nm, respectively. The hysteresis of raw Dt and CS-modified Dt at a relative pressure (P/P_0_) of 0.45–1 and 0.43–1, respectively, indicated that both samples had mesoporous properties [40]. The hysteretic loop of the type H3 in CS-modified Dt was more prominent, which might be due to the irregular slit pore formed on the surface of CS-modified Dt by the bulk heap formed by schistose CS [39].

As can be seen in Table 1, the BET surface area of CS-modified Dt, which was 148.251 m^2^/g, was 7.68 times greater than that of raw Dt (19.303 m^2^/g), which confirmed that the CS that adhered to the surface of Dt increased the specific surface area of CS-modified Dt. Meanwhile, the average pore diameter of CS-modified Dt was higher than that of raw Dt because of the modification. Given the large specific surface area and porous structure of CS-modified Dt, it could provide more adsorption sites and was a good adsorbent.

### 3.4. Zeta Analysis

**Figure 5:**
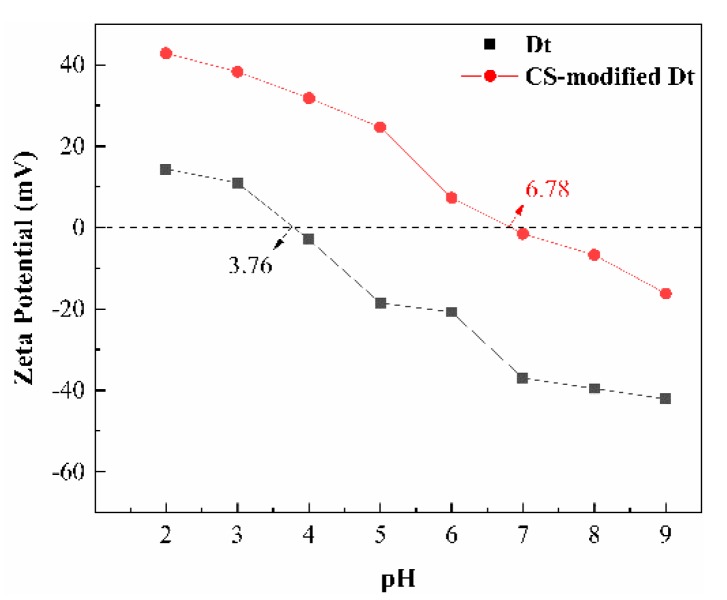
Zeta potential curves of Dt and CS-modified Dt.

As shown in Figure 5, the zeta potential of Dt and CS-modified Dt decreased with the increase of the pH value. At pH < 3.76, the silanol (–Si–OH) of Dt was protonated and attained a positive charge under the form of –Si–OH_2_^+^. By contrast, when pH > 3.76, –Si–OH_2_^+^ started to deprotonate and showed negative charge in the form of –Si–O– [41]. The pH of point of zero charge (pH_pzc_) of Dt was 3.76. The pH_pzc_ of CS-modified Dt was 6.78. As such, the amino functionalization of CS increased the pH_pzc_ of CS-modified Dt [42]. CS-modified Dt exhibited a positive potential at pH < 6.78. However, a negative potential was observed at pH > 6.78. Hence, CS-modified Dt was a modified adsorbent that had the potential to adsorb anions in acidic environment and cations in the alkaline condition [39].

### 3.5. XPS Analysis

**Figure 6:**
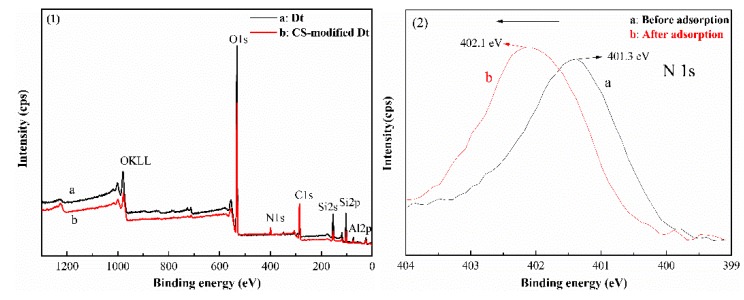
XPS spectra of (1)a Dt, (1)b CS-modified Dt, (2)a N1s energy before adsorption, and (2)b N1s energy after adsorption.

X-ray photoelectron spectroscopy (XPS) wide-scan spectra of the Dt and CS-modified Dt are respectively shown in Figure 6((1)a,(1)b). The signals with binding energies at 399.1 and 285.5 eV, attributable to the N 1s and C 1s species respectively [43,44], appeared in the XPS wide scan spectrum of the CS-modified Dt, clearly suggesting the successful immobilization of CS on the surface of Dt [45]. As shown in Figure 6((2)a), the peak at approximately 401.3 eV corresponds to the protonated N in CS (Ncs) [46]. In addition, the binding energy (BE) of Ncs is shifted to higher values (Figure 6((2)b), BE = 402.1 eV) after As(V) adsorption. This finding indicates that the Ncs play an indispensable role in arsenic adsorption [47].

### 3.6. XRD Analysis

**Figure 7:**
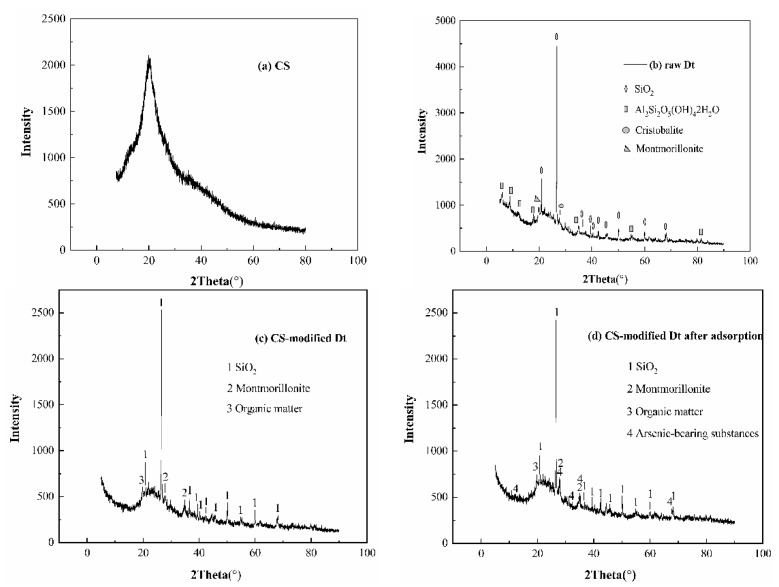
XRD patterns of (**a**) CS, (**b**) raw Dt, (**c**) CS-modified Dt, and (**d**) CS-modified Dt after adsorption.

Figure 7 shows the X-ray powder diffraction pattern of (a) CS, (b) raw Dt, (c) CS-modified Dt, and (d) CS-modified Dt after adsorption. Figure 7a suggests that CS exhibited typical semi-crystalline properties (110) at its diffraction peak at 19.92° [JCPDS File No. 35-1974] [48]. Figure 7b suggests that the raw Dt consists mainly of silica (SiO_2_) [49] with a little kaolin (Al_2_Si_2_O_5_(OH)_4_2H_2_O) [50], cristobalite [51], and montmorillonite [52]. The diffraction broad-bands observed in range of 18–32° may be attributed to the amorphous SiO_2_ [53]. Comparing Figure 7b,c, the crystalline characteristic peak intensity of both Dt is much reduced, the characteristic diffraction peaks of cristobalite is almost disappeared, and the characteristic diffraction peak of CS (2θ = 19.92°) appears, which indicated that CS and Dt have formed a good interface attachment effect [39]. Figure 7d shows that the peak position of CS remains a constant except that the intensity of characteristic diffraction peak is slightly increased, indicating that the structure of CS-modified Dt has a certain stability, and the diffraction peak of arsenic-bearing substances appears, indicating that the CS has a certain adsorption effect on arsenic.

### 3.7. Adsorption Analysis

#### 3.7.1. Effect of Adsorbent Dosage

**Figure 8:**
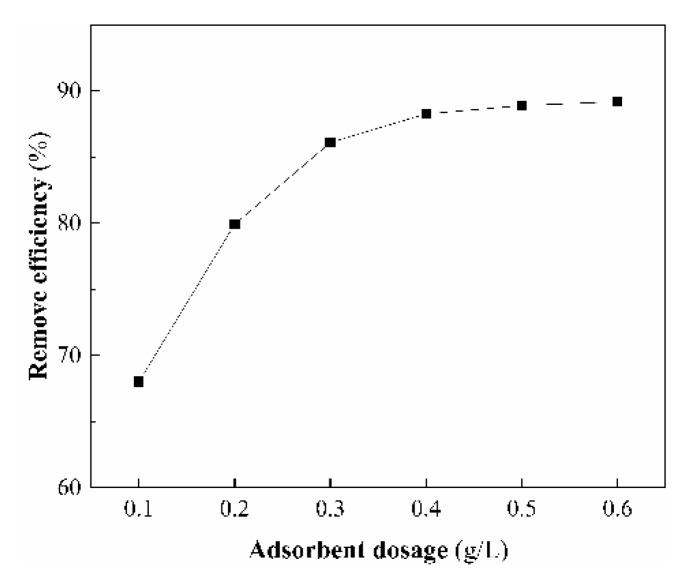
Effect of adsorbent dosage on the As(V) removal.

The adsorbent dosage determines the removal efficiency of an adsorbent in a fixed initial concentration of adsorbent. The effect of adsorbent amount on As(V) removal was investigated by changing the dosage from 0.1 to 0.6 g/L at initial As(V) concentration of 5 mg/L. The results are shown in Figure 8 and as exhibits, by increasing the dose of CS-modified Dt, the removal of As(V) increased from 68.0% to 89.2%. It is noticed that the As(V) removal efficiency increased rapidly as the adsorbent dosage increased from 0.1 to 0.4 g/L, which may be due to that the active sites for binding target ions on the adsorbent surface was less at a lower dose of adsorbent [54]. When the dosage of the CS-modified Dt was more than 0.4 g/L, the removal efficiency increased relatively slowly, and the adsorption surface of CS-modified Dt might gradually reach the saturation state [35]. Therefore, considering As(V) removal and economy, 0.4 g/L is the optimal value of adsorbent dosage.

#### 3.7.2. Effect of pH

**Figure 9:**
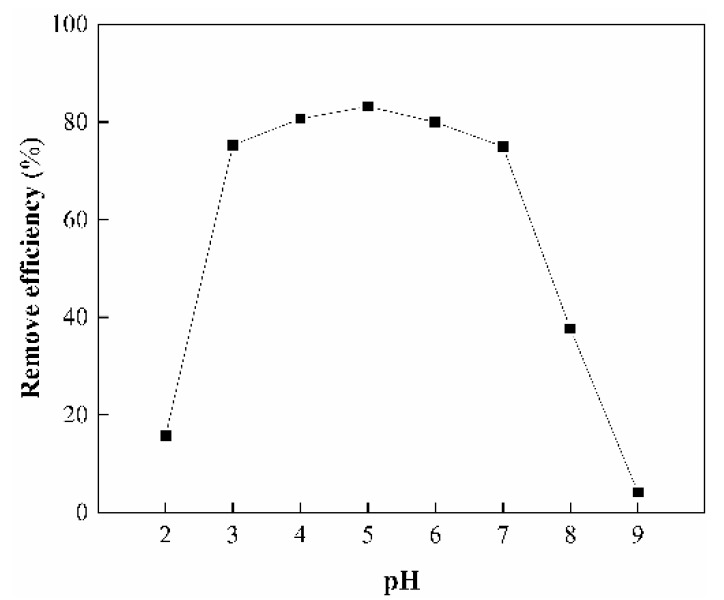
Effect of initial pH on the As(V) removal.

**Figure 10:**
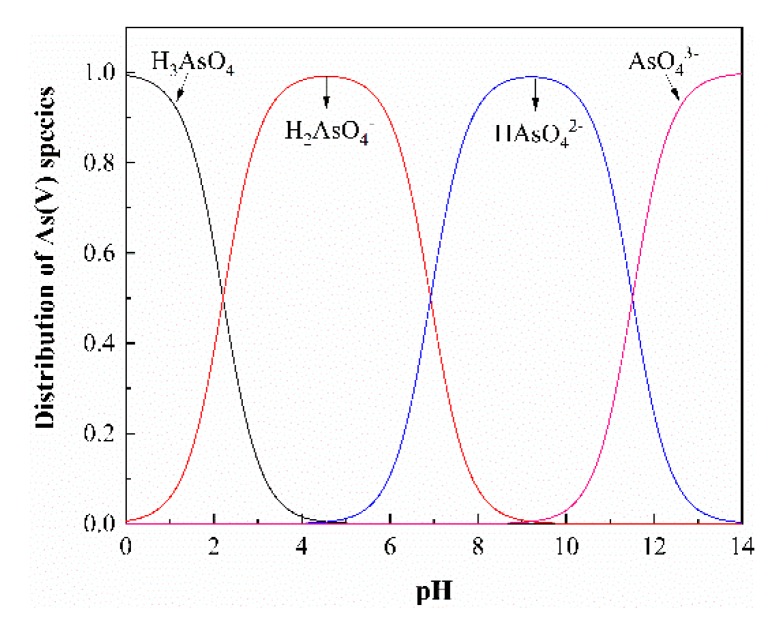
Distribution of As(V) species as a function of pH based on the equilibrium constants.

The pH plays an important role on adsorbing metal ions, since it determines the degree of ionization and the surface charge of the adsorbent as well as the species of the adsorbate [55]. The effect of pH was investigated over a broad pH range of 2–9 with the dosage of adsorbent at 0.4 g/L and As(V) initial concentration 5 mg/L at room temperature (298 K). The results were shown in Figure 9. In the pH range of 2–3, the removal efficiency increases sharply as the pH increasing. The removal efficiency increases slightly at pH 3–5, decreases slightly at pH 5–7, and then decreases sharply at pH 7–9. The different removal behavior of As(V) on adsorbent can be explained by the distribution forms of As(V) at various pH values in Figure 10. As can be seen, As(V) exists mainly as neutral H_3_AsO_4_ species at pH 2, only physical adsorption driving forces between H_3_AsO_4_ and the sorbent were present, resulting in less adsorption. In the range of pH 3–7, the predominant species is H_2_AsO_4_^−^. The amount of H_2_AsO_4_^−^ reaches the maximum at pH 5, which leads to the strongest electrostatic interaction between H_2_AsO_4_^−^ and protonated-amino groups on the surface of CS-modified Dt [56], so that the removal efficiency reaches 83.2%. At pH 7–9, the main species are H_2_AsO_4_^−^ and HAsO_4_^2−^, hence, the removal efficiency of As(V) decrease can be contributed partly to the competition between OH^−^ and negative charged As(V) anions (H_2_AsO_4_^−^, HAsO_4_^2−^) [57]. The H_2_AsO_4_^−^ and HAsO_4_^2−^ are difficult to be adsorbed on the surface of amino-deprotonated CS-modified Dt at higher pH values [40]. Therefore, the optimum pH for As(V) removal is 5.

#### 3.7.3. Effect of Contact Time

**Figure 11:**
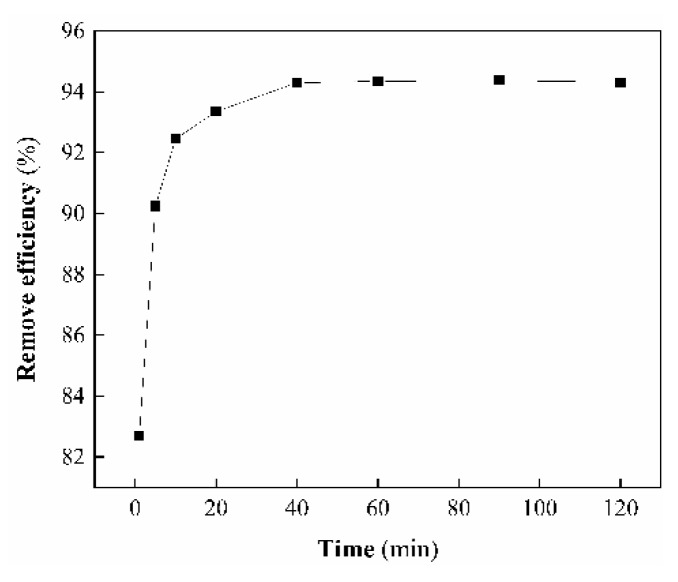
Effect of contact time on the As(V) removal.

The effect of contact time on the removal efficiency of As(V) was investigated by varying the contact times from 2 to 120 min under the following conditions: 5 mg/L initial As(V) concentration, 0.4 g/L sorbent dosage, and temperature set at 298 K. As clearly seen from Figure 11, the removal efficiency of As(V) was rapid within the first 10 min, due to more availability adsorption sites and smaller mass transfer resistance on the adsorbent surface, and slowly increased with time until it reached saturation. The adsorption reached equilibrium in about 40 min. The observed removal efficiency was 94.3% at 40 min. The differences in the adsorption values after 40 min were very small. So, this duration was selected as the optimum contact time.

#### 3.7.4. Adsorption Isotherm

**Figure 12:**
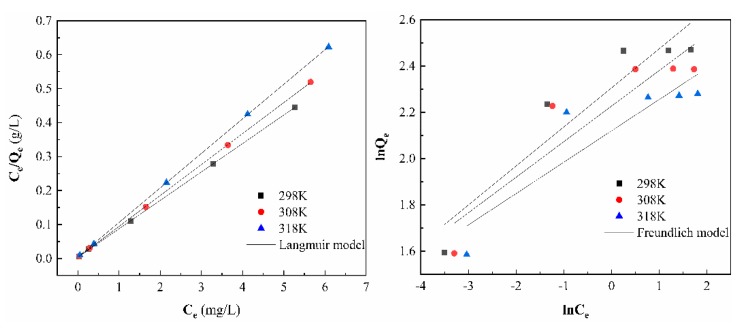
Adsorption isotherms for As(V) adsorption by CS-modified Dt (sorbent dosage: 0.4 g/L; contact Table. 40 min; pH 5).

In this work, the adsorption isotherms were obtained for the sorption of As(V) ions onto the CS-modified Dt by altering the initial concentration of the solution in the range of 2–10 mg/L under the optimized batch conditions. The linear fit of isotherms of Langmuir and Freundlich can be seen from Figure 12 and Table 2, the adsorption of As(V) by CS-modified Dt is better fitted by Langmuir model as correlation coefficients (R^2^ > 0.99) were higher compared to Freundlich throughout the temperature ranges. Apparently, the adsorption occurred on the CS-modified Dt surface by monomolecular layer sorption [58]. Furthermore, the values of *Q_m_* decreased with temperature increase which is in line with theoretical adsorption capacities of CS-modified Dt which ranged from 11.95 mg/g at 298 K to 9.83 mg/g at 318 K, which indicate the exothermic route of As(V) sorption onto CS-modified Dt [54].

#### 3.7.5. Adsorption Kinetics

**Figure 13:**
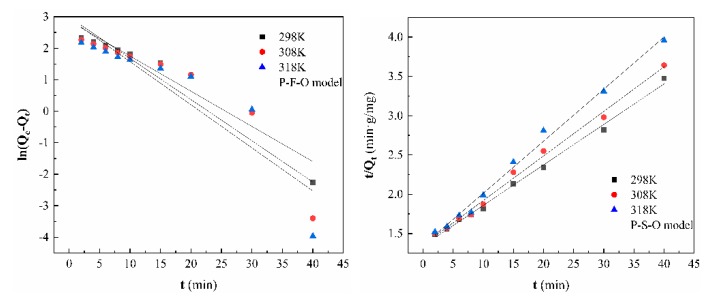
Adsorption kinetic for As(V) adsorption by CS-modified Dt (sorbent dosage: 0.4 g/L; initial As(V) ions concentration: 5 mg/L; pH 5).

The linear fit of kinetics of PFO and PSO can be seen from Figure 13 and Table 3, the PSO (R^2^ > 0.99) has a better fit than that of PFO model for the adsorption of As(V). Therefore, the adsorption data were well obeyed PSO kinetic model, which indicated that the adsorption rate mainly depended on chemisorption with exchange of ions on the arsenate and the protonated-amino [59]. The adsorption mechanism is expressed as follows:

(9) $$\left(\mathrm{Dt}@\mathrm{CS}\right)–{\mathrm{NH}}_{2}+H_{2}O⇌\left(\mathrm{Dt}@\mathrm{CS}\right)–{\mathrm{NH}}_{3}^{+}{\mathrm{OH}}^{-},$$

(10) $$\begin{array}{c} \left(\mathrm{Dt}@\mathrm{CS}\right)–{\mathrm{NH}}_{3}^{+}{\mathrm{OH}}^{-}+{\mathrm{Na}}^{+}H_{n}{\mathrm{AsO}}_{4}^{\left(3-n\right)-}⇌\left(\mathrm{Dt}@\mathrm{CS}\right)–{\mathrm{NH}}_{3}^{+}H_{n}{\mathrm{AsO}}_{4}^{\left(3-n\right)-}+\\ {\mathrm{Na}}^{+}{\mathrm{OH}}^{-}\;\left(n=1,\;2,\;3\right). \end{array}$$

### 3.8. Desorption and Regeneration Analysis

**Figure 14:**
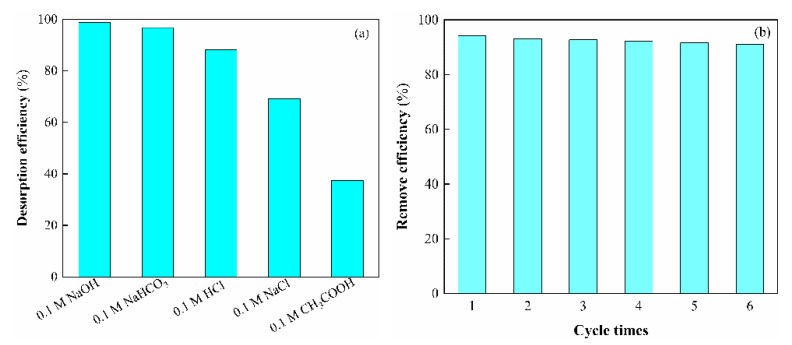
(**a**) Effect of type of desorption solvent on the desorption rate. (**b**) The reusability of CS-modified Dt for removal of As(V) ions.

Desorption of the adsorbed As(V) anions from the CS-modified Dt was also studied in a batch experimental system. Various factors are probably involved in determining efficiencies of As(V) desorption, such as the extent of hydration of the metal ions and sorbent microstructure. However, an important factor appears to be binding strength. Figure 14a illustrates As(V) desorption with NaOH, NaHCO_3_, HCl, NaCl, and CH_3_COOH. According to results, 0.1 M NaOH showed the best desorption efficiency among all the solvents. In an alkaline solution the surface charge of CS-modified Dt possibly changed thereby causing detachment of the arsenic ions from the adsorbent. To show the reusability of the CS-modified Dt, adsorption-desorption cycle was repeated 6 times by using the same CS-modified Dt. As shown in Figure 14b, the removal efficiency still remained as 90.98% after six cycles. The result indicated that the prepared CS-modified Dt could be effectively used for wastewater treatment containing As(V) ions at least for 6 times.

## 4. Conclusions

Chitosan (CS) is a great potential adsorbent for wastewater treatment. In this study, a novel CS-modified Dt sorbent was prepared and characterized by using SEM, FTIR, and XRD techniques, and the CS-modified Dt successfully applied as a novel biocomposite sorbent for the removal of As(V) ions from aqueous solution using the batch method. The maximum removal rate of the As(V) ions reached 94.3% with the optimum adsorbent dosage of 0.4 g/L, pH 5, and 40 min contact time at 298 K. It was found that the adsorption experiment data of CS-modified Dt fit well to be the P–S–O kinetic model and Langmuir isotherm model, and the adsorption process of the As(V) ions onto the CS-modified Dt was based on monolayer chemical adsorption. Furthermore, the desorption efficiency reached 98.76% with 0.1 M NaOH as the desorbent. Most importantly, the data obtained in this study show that the removal efficiency of As(V) shows no significant decrease after six adsorption-desorption cycles. In summary, CS-modified Dt will be an environmental and cost-effective adsorbent applied in the fields of As(V) ions adsorption with strong removal efficiency and excellent stability.

## Figures and Tables

**Table 1:** Comparison of Brunauer–Emmett–Teller (BET) surface area and BJH (Barrett–Joyner–Halenda) average pore diameter of raw Dt and CS-modified Dt.

Sample	BET Surface Area (m^2^/g)	BJH Average Pore Diameter (nm)
Dt	19.303	9.998
CS-modified Dt	148.251	5.185

**Table 2:** Parameters of the Langmuir and Freundlich equation.

T(K)	Langmuir			Freundlich		
	Q_m_	K_L_	R^2^	1/n	K_F_	R^2^
298	11.95	21.88	0.9999	0.17	10.03	0.8426
308	10.98	25.92	0.9999	0.15	9.27	0.7970
318	9.83	22.37	0.9999	0.14	8.33	0.7467

**Table 3:** Fitting parameters of kinetic equation.

T(K)	PFO			PSO		
	Q_e_	K_1_	R^2^	Q_e_	K_2_	R^2^
298	19.83	0.131	0.8536	19.40	0.002	0.9948
308	18.94	0.137	0.8076	17.63	0.002	0.9937
318	17.58	0.112	0.9241	15.08	0.003	0.9922

## References

[1] Dong Z., Liu C.X., Liu Y., Yan K., Semple K.T., Naidu R. (2016). Using publicly available data, a physiologically-based pharmacokinetic model and bayesian simulation to improve arsenic non-cancer dose-response. Environ. Int..

[2] Yan K., Naidu R., Liu Y., Wijayawardena A., Duan L., Dong Z. (2018). A pooled data analysis to determine the relationship between selected metals and arsenic bioavailability in soil. Int. J. Environ. Res. Public Health.

[3] Yang Q., Li Z., Lu X., Duan Q., Huang L., Bi J. (2018). A review of soil heavy metal pollution from industrial and agricultural regions in China: Pollution and risk assessment. Sci. Total Environ..

[4] Kumari V., Bhaumik A. (2015). Mesoporous ZnAl_2_O_4_: An efficient adsorbent for the removal of arsenic from contaminated water. Dalton. Trans..

[5] Sineephan T., Apichat I., Narong P. (2016). Iron-loaded zein beads as a biocompatible adsorbent for arsenic (V) removal. J. Ind. Eng. Chem..

[6] Sharma A.K., Tjell J.C., Sloth J.J., Holm P.E. (2014). Review of arsenic contamination, exposure through water and food and low cost mitigation options for rural areas. Appl. Geochem..

[7] Katsoyiannis I.A., Tzollas N.M., Tolkou A.K., Mitrakas M., Ernst M., Zouboulis A.I. (2017). Use of novel composite coagulants for arsenic removal from waters-experimental insight for the application of polyferric sulfate (PFS). Sustainability.

[8] Katsoyiannis I.A., Voegelin A., Zouboulis A.I., Hug S.J. (2015). Enhanced As(III) oxidation and removal by combined use of zero valent iron and hydrogen peroxide in aerated waters at neutral pH values. J. Hazard. Mater..

[9] Fogarassy E., Galambos I., Bekassy M.E., Vatai G. (2009). Treatment of high arsenic content wastewater by membrane filtration. Desalination.

[10] Zhao D., Yu Y., Chen J.P. (2016). Fabrication and testing of zirconium-based nanoparticle-doped activated carbon fiber for enhanced arsenic removal in water. RSC Adv..

[11] Hering J.G., Katsoyiannis I.A., Theoduloz G.A., Berg M., Hug S.J. (2017). Arsenic removal from drinking water: Experiences with technologies and constraints in practice. J. Environ. Eng..

[12] Zhu L.Y., Zhu Z.L., Qiu Y.L., Zhang R.H. (2014). Synthesis of As (V)-Cr (III) co-imprinted polymer and its adsorption performance for arsenate species. Sep. Sci. Technol..

[13] Kumar M.N.V.R. (2000). A review of chitin and chitosan applications. React. Funct. Polym..

[14] Jiang C., Wang X., Wang G., Hao C., Li X., Li T. (2019). Adsorption performance of a polysaccharide composite hydrogel based on crosslinked glucan/chitosan for heavy metal ions. Compos. Part B Eng..

[15] Liu Y., Wang R., Bai J., Jiao T., Bai Z., Zhang L., Zhang Q., Zhou J., Peng Q. (2019). Non-covalent self-assembly of multi-target polystyrene composite adsorbent with highly efficient Cu (II) ion removal capability. Colloid. Surf. A.

[16] Yuvaraja G., Pang Y., Chen D.Y., Kong L.J., Mehmood S., Subbaiah M.V., Rao D.S., Pavuluri C.M., Wen J.C., Reddy G.M. (2019). Modification of chitosan macromolecule and its mechanism for the removal of Pb(II) ions from aqueous environment. Int. J. Biol. Macromol..

[17] Jamshidifard S., Koushkbaghi S., Hosseini S., Rezaei S., Karamipour A., Jafarirad A., Irani M. (2019). Incorporation of UiO-66-NH_2_ MOF into the PAN/chitosan nanofibers for adsorption and membrane filtration of Pb(II), Cd(II) and Cr(VI) ions from aqueous solutions. J. Hazard. Mater..

[18] Wei Y., Yu X., Liu C., Ma J., Wei S., Chen T., Yin K., Liu H., Luo S. (2019). Enhanced arsenite removal from water by radially porous Fe-chitosan beads: Adsorption and H_2_O_2_ catalytic oxidation. J. Hazard. Mater..

[19] Correa M.M.A., López C.J., Sánchez M.D.I., Sánchez D.R.G. (2014). Synthesis and application of modified chitosan beads for iron removal: Kinetic and isotherm models. Asia-Pac. J. Chem. Eng..

[20] Fan D., Zhu X., Xu M., Yan J. (2006). Adsorption properties of chromium (VI) by chitosan coated montmorillonite. J. Biol. Sci..

[21] Futalan C.M., Kan C.C., Dalida M.L., Hsien K.J., Pascua C., Wan M.W. (2011). Comparative and competitive adsorption of copper, lead, and nickel using chitosan immobilized on bentonite. Carbohydr. Polym..

[22] Wan M.W., Kan C.C., Rogel B.D., Dalida M.L.P. (2010). Adsorption of copper(II) and lead(II) ions from aqueous solution on chitosan-coated sand. Carbohydr. Polym..

[23] Boddu V.M., Abburi K., Randolph A.J., Smith E.D. (2008). Removal of copper(II) and nickel(II) ions from aqueous solutions by a composite chitosan biosorbent. Sep. Sci. Technol..

[24] Boddu V.M., Abburi K., Talbott J.L., Smith E.D., Haasch R. (2008). Removal of arsenic(III) and arsenic(V) from aqueous medium using chitosan-coated biosorbent. Water. Res..

[25] Šljivić M., Smičiklas I., Pejanović S., Plećaš I. (2009). Comparative study of Cu^2+^ adsorption on a zeolite, a clay and a diatomite from Serbia. Appl. Clay Sci..

[26] Sari A., Çıtak D., Tuzen M. (2010). Equilibrium, thermodynamic and kinetic studies on adsorption of Sb (III) from aqueous solution using low-cost natural diatomite. Chem. Eng. J..

[27] Zhao P., Zhang R., Wang J. (2017). Adsorption of methyl orange from aqueous solution using chitosan/diatomite composite. Water. Sci. Technol..

[28] Etemadi M., Samadi S., Yazd S.S., Jafari P., Yousefi N., Aliabadi M. (2017). Selective adsorption of Cr(VI) ions from aqueous solutions using Cr^6+^-imprinted Pebax/chitosan/GO/APTES nanofibrous adsorbent. Int. J. Biol. Macromol..

[29] Mu Y., Cui M., Zhang S., Zhao J., Meng C., Sun Q. (2018). Comparison study between a series of new type functional diatomite on methane adsorption performance. Micropor. Mesopor. Mater..

[30] Fan H.T., Fan X., Li J., Guo M., Zhang D., Yan F., Sun T. (2012). Selective removal of arsenic (v) from aqueous solution using a surface-ion-imprinted amine-functionalized silica gel sorbent. Ind. Eng. Chem. Res..

[31] Song X., Li L., Geng Z., Zhou L., Ji L. (2017). Effective and selective adsorption of As(III) via imprinted magnetic Fe_3_O_4_/HTCC composite nanoparticles. J. Environ. Chem. Eng..

[32] Qi X., Gao S., Ding G., Tang A.N. (2017). Synthesis of surface Cr (VI)-imprinted magnetic nanoparticles for selective dispersive solid-phase extraction and determination of Cr (VI) in water. Talanta.

[33] Zhang Y.Z., Li J., Li W.J., Li Y. (2015). Adsorption of sunset yellow FCF from aqueous solution by chitosan-modified diatomite. Water. Sci. Technol..

[34] Bayramoglu G., Arica M.Y. (2011). Synthesis of Cr (VI)-imprinted poly (4-vinyl pyridine-co-hydroxyethyl methacrylate) particles: Its adsorption propensity to Cr (VI). J. Hazard. Mater..

[35] Fu Y., Huang Y., Hu J., Zhang Z. (2018). Preparation of chitosan/amine modified diatomite composites and adsorption properties of Hg (II) ions. Water. Sci. Technol..

[36] Zhang L., Xue J., Zhou X., Fei X., Wang Y., Zhou Y., Zhong L., Han X. (2014). Adsorption of molybdate on molybdate-imprinted chitosan/triethanolamine gel beads. Carbohydr. Polym..

[37] Wong Y.C., Szeto Y.S., Cheung W.H., Mckay G. (2003). Equilibrium studies for acid dye adsorption onto chitosan. Langmuir.

[38] Chen J.H., Li G.P., Liu Q.L., Ni J.C., Wu W.B., Lin J.M. (2010). Cr (III) ionic imprinted polyvinyl alcohol/sodium alginate (PVA/SA) porous composite membranes for selective adsorption of Cr (III) ions. Chem. Eng. J..

[39] Song X., Chai Z., Zhu Y., Li C., Liang X. (2019). Preparation and characterization of magnetic chitosan-modified diatomite for the removal of gallic acid and caffeic acid from sugar solution. Carbohydr. Polym..

[40] Yan L.G., Yang K., Shan R.R., Yan T., Wei J., Yu S.J., Yu H.Q., Du B. (2015). Kinetic, isotherm and thermodynamic investigations of phosphate adsorption onto core–shell Fe_3_O_4_@LDHs composites with easy magnetic separation assistance. J. Colloid. Interface Sci..

[41] Sheng G., Wang S., Hu J., Lu Y., Li J., Dong Y., Wang X. (2009). Adsorption of Pb(II) on diatomite as affected via aqueous solution chemistry and temperature. Colloid. Surf. A.

[42] Gao B., Li X., Chen T., Fang L. (2014). Preparation of molybdate anion surface-imprinted material for selective removal of molybdate anion from water medium. Ind. Eng. Chem. Res..

[43] Janciauskaite U., Rakutyte V., Miskinis J., Makuska R. (2008). Synthesis and properties of chitosan-*N*-dextran graft copolymers. React. Funct. Polym..

[44] Simsek E.B., Saloglu D., Ozcan N., Novak I., Berek D. (2017). Carbon fiber embedded chitosan/PVA composites for decontamination of endocrine disruptor bisphenol-A from water. J. Taiwan Inst. Chem. Eng..

[45] Wang J., Han Y., Li J., Wei J. (2017). Selective adsorption of thiocyanate anions using straw supported ion imprinted polymer prepared by surface imprinting technique combined with RAFT polymerization. Sep. Purif. Technol..

[46] Vieira R.S., Oliveira M.L.M., Guibal E., Rodríguez C.E., Beppu M.M. (2011). Copper, mercury and chromium adsorption on natural and crosslinked chitosan films: An XPS investigation of mechanism. Colloid. Surf. A.

[47] Fang L., Min X., Kang R., Yu H., Pavlostathis S.G., Luo X. (2018). Development of an anion imprinted polymer for high and selective removal of arsenite from wastewater. Sci. Total Environ..

[48] Kumar I.A., Viswanathan N. (2018). Development and reuse of amine-grafted chitosan hybrid beads in the retention of nitrate and phosphate. J. Chem. Eng. Data.

[49] Awadh S.M., Yaseen Z.M. (2019). Investigation of silica polymorphs stratified in siliceous geode using FTIR and XRD methods. Mater. Chem. Phys..

[50] Prasanphan S., Wannagon A., Kobayashi T. (2019). Reaction mechanisms of calcined kaolin processing waste-based geopolymers in the presence of low alkali activator solution. Constr. Build. Mater..

[51] Musyarofah, Soontaranon S., Limphirat W., Triwikantoro, Pratapa S. (2019). XRD, WAXS, FTIR, and XANES studies of silica-zirconia systems. Ceram. Int..

[52] Zadaka A.D., Bleiman N., Mishael Y.G. (2013). Sepiolite as an effective natural porous adsorbent for surface oil-spill. Micropor. Mesopor. Mater..

[53] Das H., Debnath N., Arai T., Arai T., Kawaguchi T., Sakamoto N., Shinozaki K., Suzuki H., Wakiya N. (2019). Superparamagnetic magnesium ferrite/silica core-shell nanospheres: A controllable SiO_2_ coating process for potential magnetic hyperthermia application. Adv. Powder Technol..

[54] Caner N., Sarı A., Tüzen M. (2015). Adsorption characteristics of mercury (II) ions from aqueous solution onto chitosan-coated diatomite. Ind. Eng. Chem. Res..

[55] Lasheen M.R., Ammar N.S., Ibrahim H.S. (2012). Adsorption/desorption of Cd (II), Cu (II) and Pb (II) using chemically modified orange peel: Equilibrium and kinetic studies. Solid. State Sci..

[56] Chen D., Huang C., He M., Hu B. (2009). Separation and preconcentration of inorganic arsenic species in natural water samples with 3-(2-aminoethylamino) propyltrimethoxysilane modified ordered mesoporous silica micro-column and their determination by inductively coupled plasma optical emission spectrometry. J. Hazard. Mater..

[57] Mustafai F.A., Balouch A., Abdullah., Jalbani N., Bhanger M.I., Jagirani M.S., Kumar A., Tunio A. (2018). Microwave-assisted synthesis of imprinted polymer for selective removal of arsenic from drinking water by applying Taguchi statistical method. Eur. Polymer. J..

[58] Sun L., Chen D., Wan S., Yu Z. (2015). Performance, kinetics, and equilibrium of methylene blue adsorption on biochar derived from eucalyptus saw dust modified with citric, tartaric, and acetic acids. Bioresour. Technol..

[59] Kyzas G.Z., Bikiaris D.N. (2015). Recent modifications of chitosan for adsorption applications: A critical and systematic review. Mar. Drugs.

